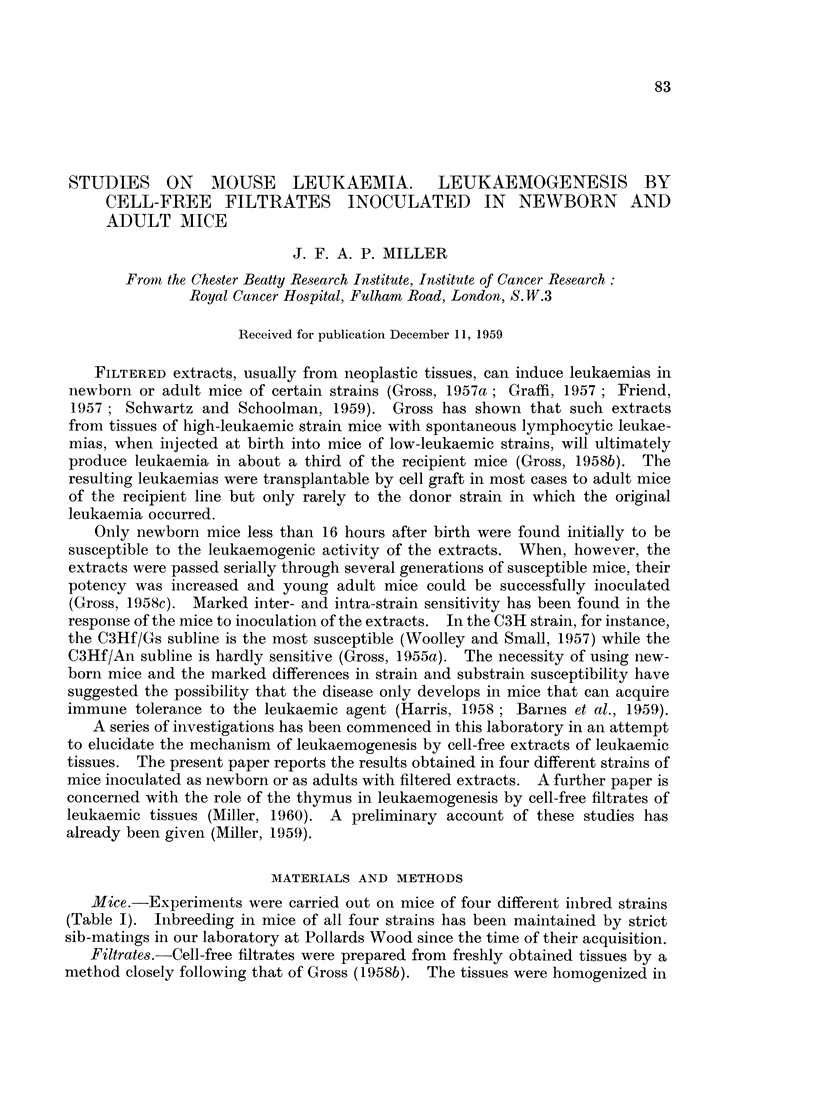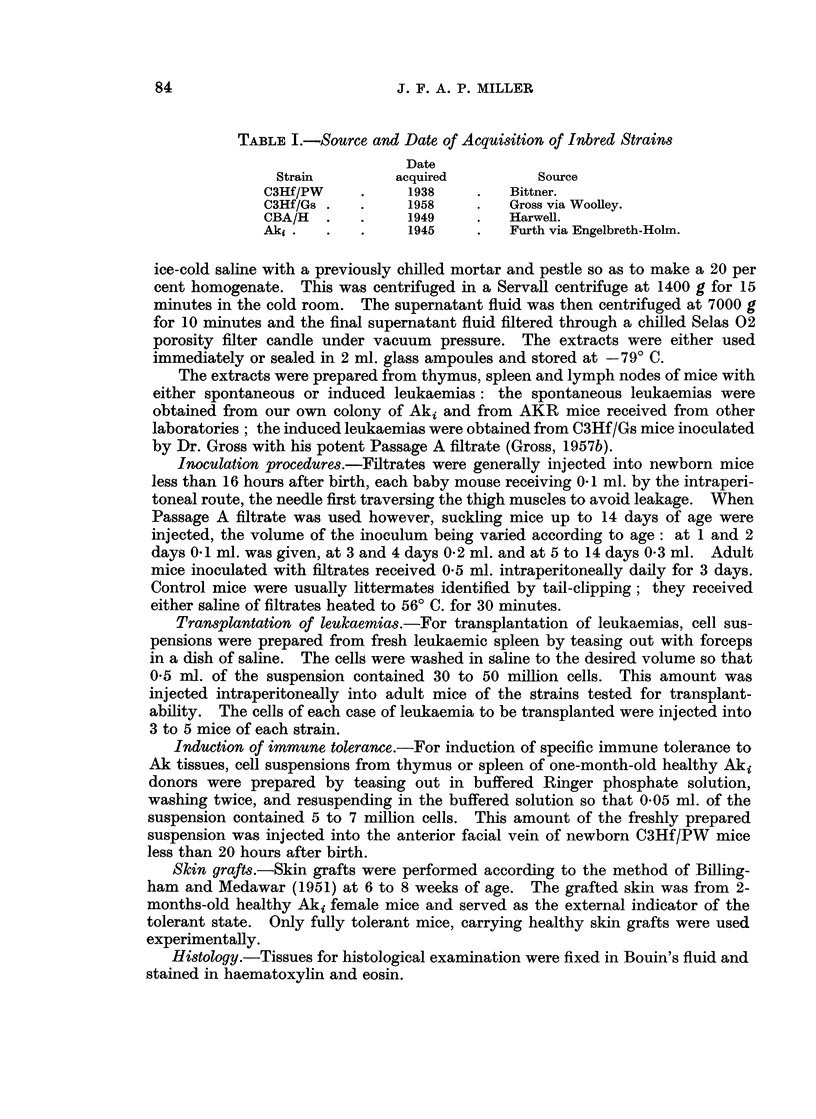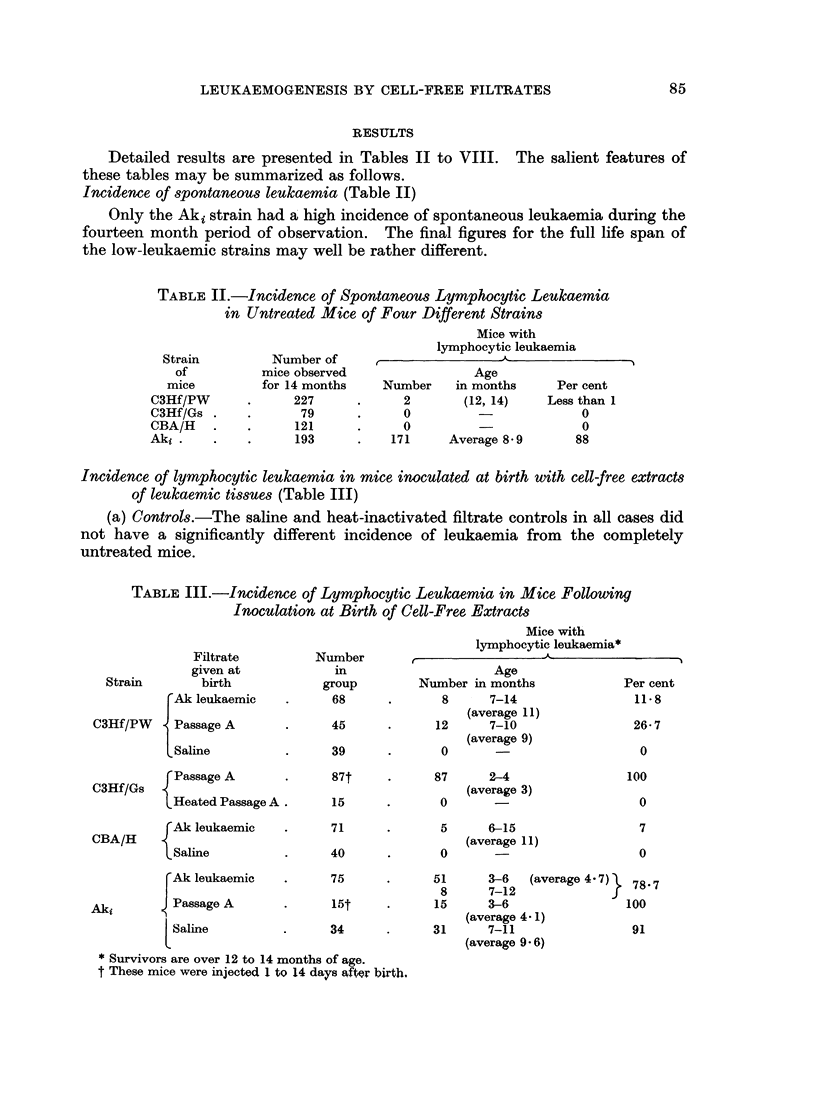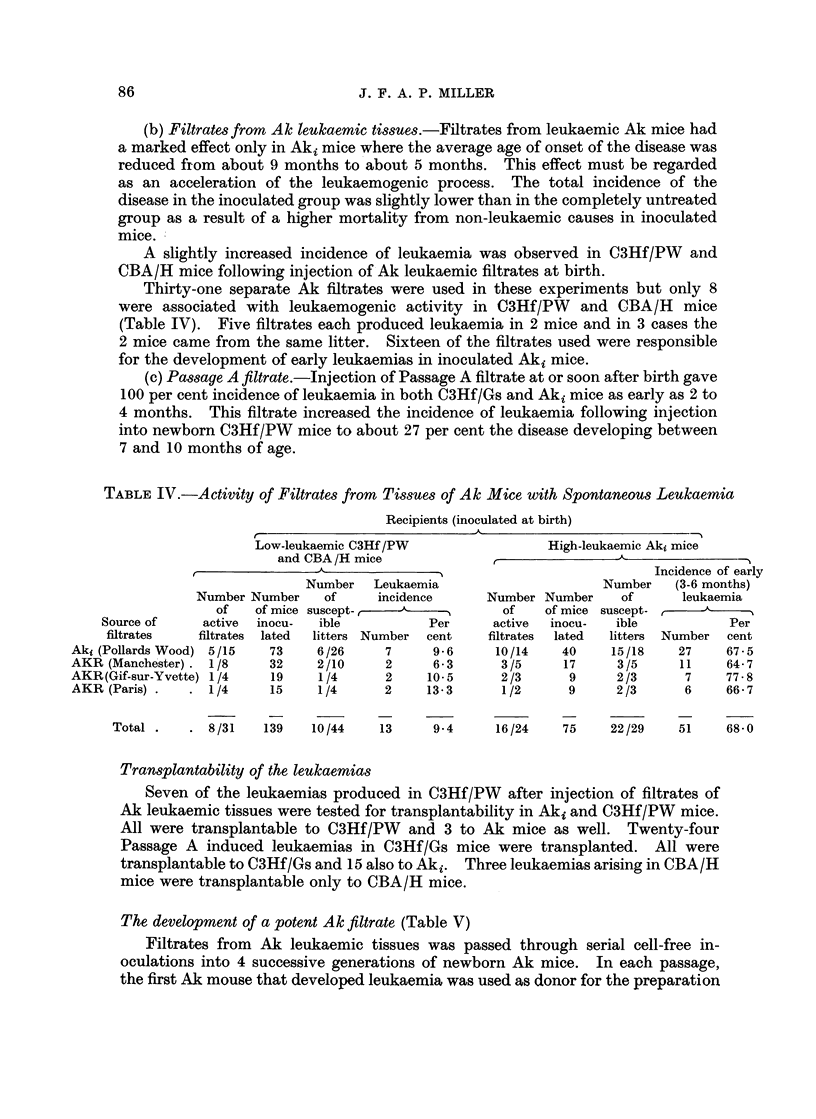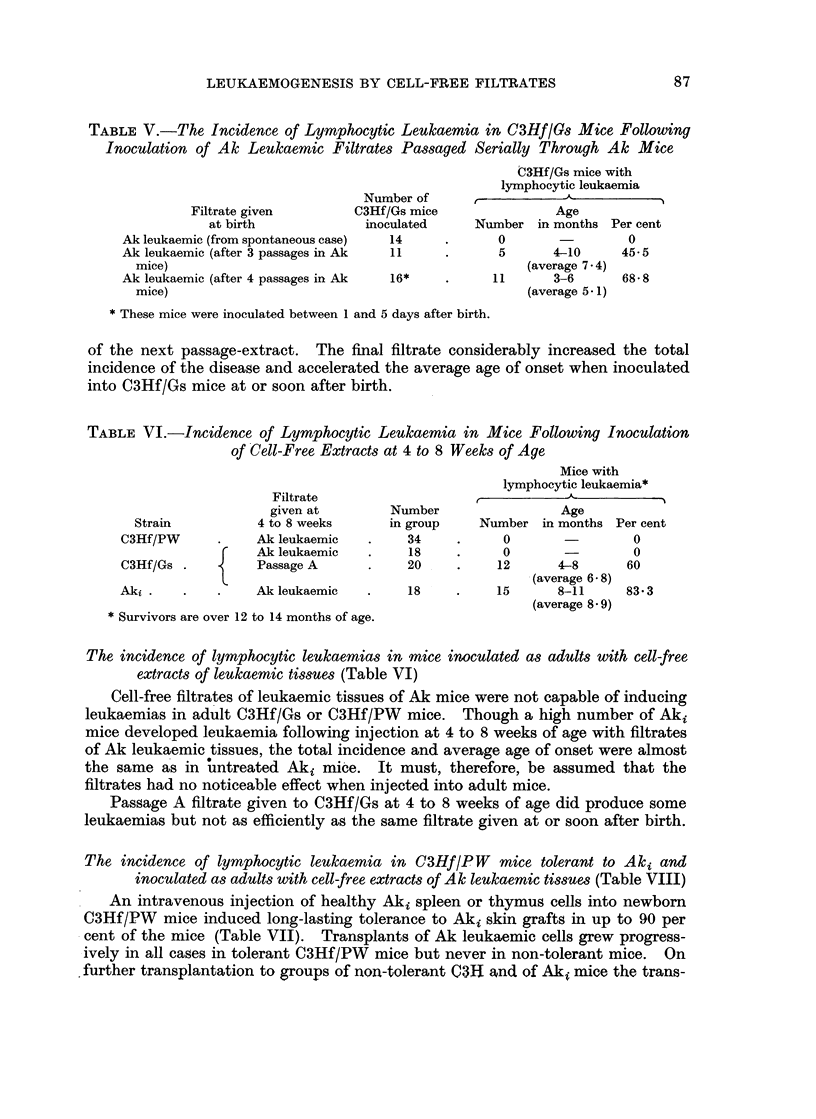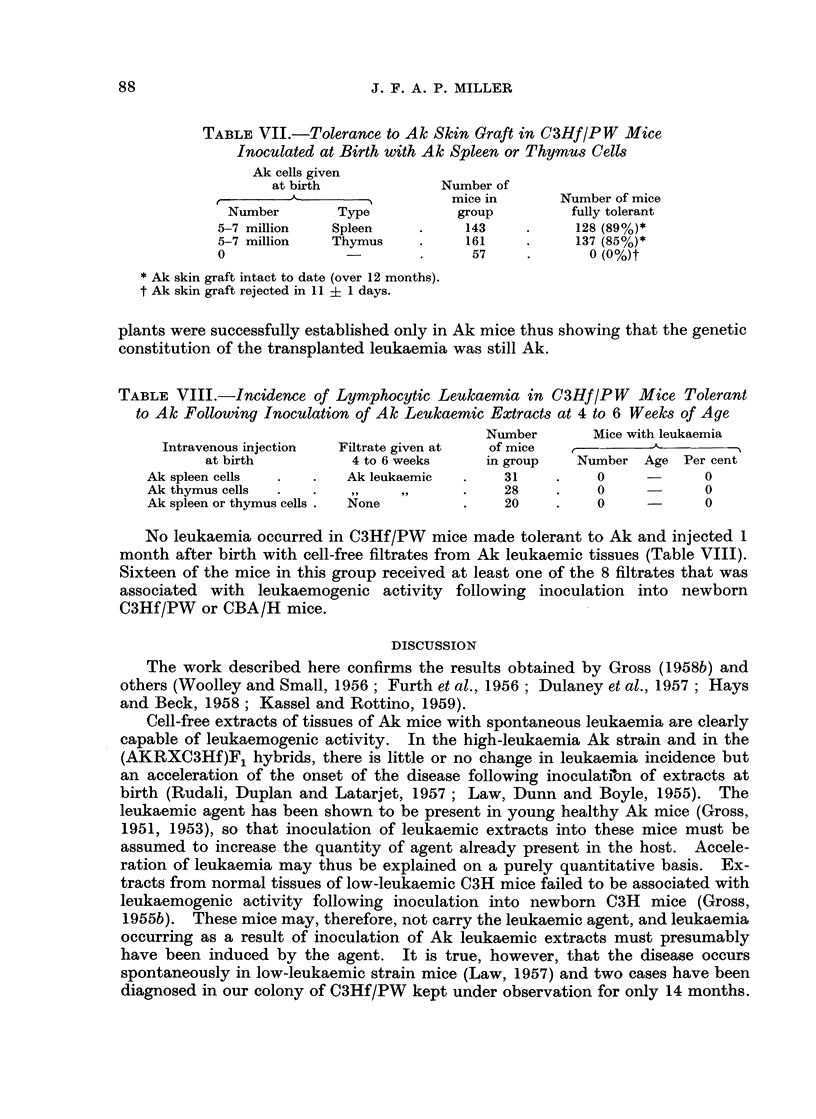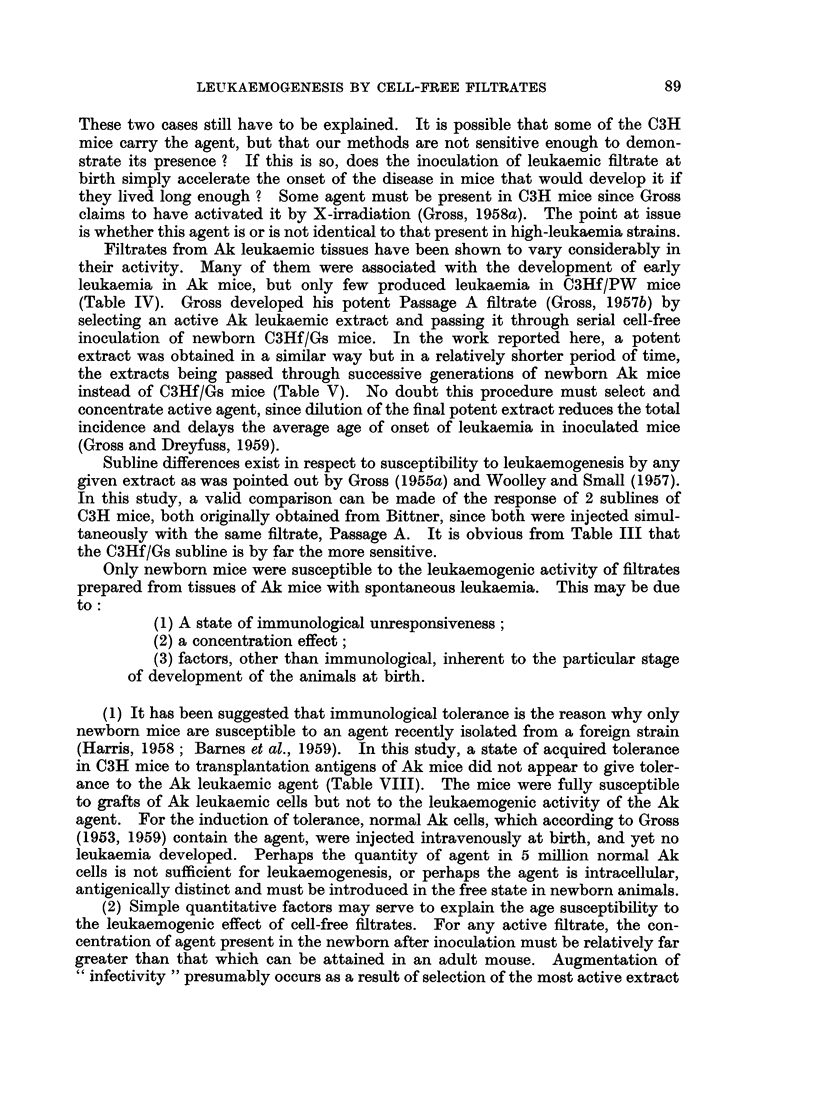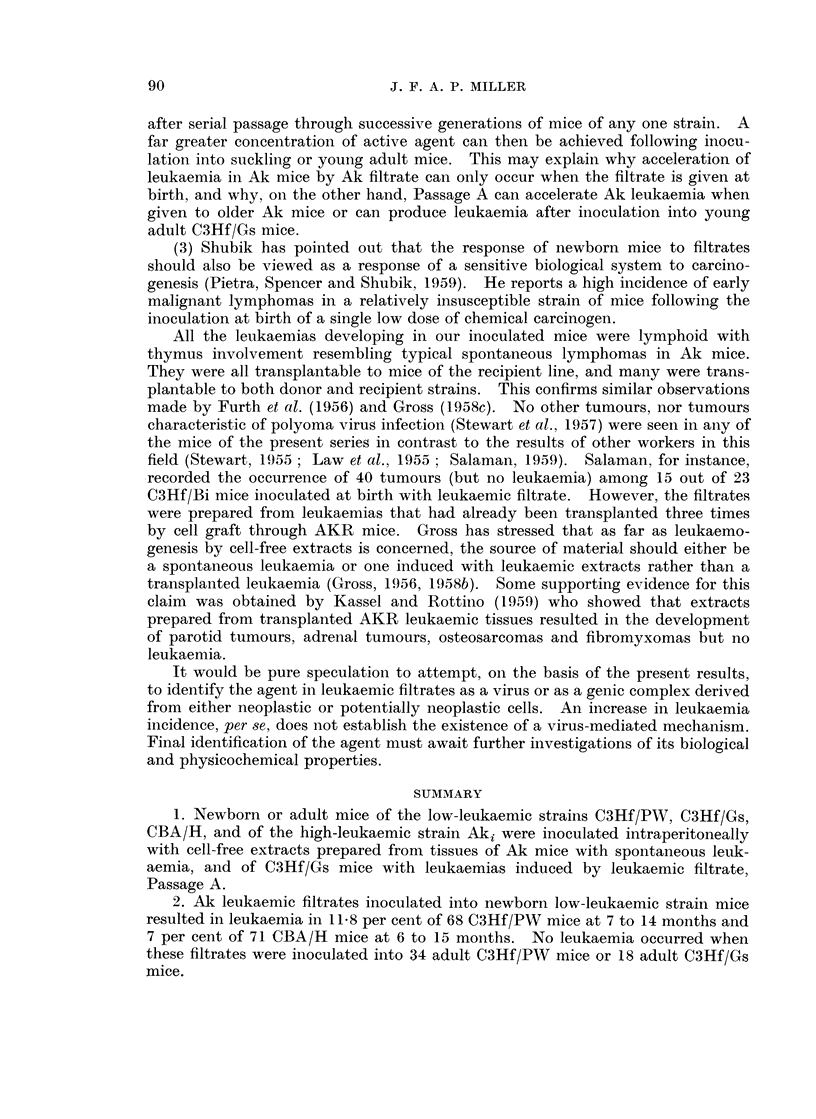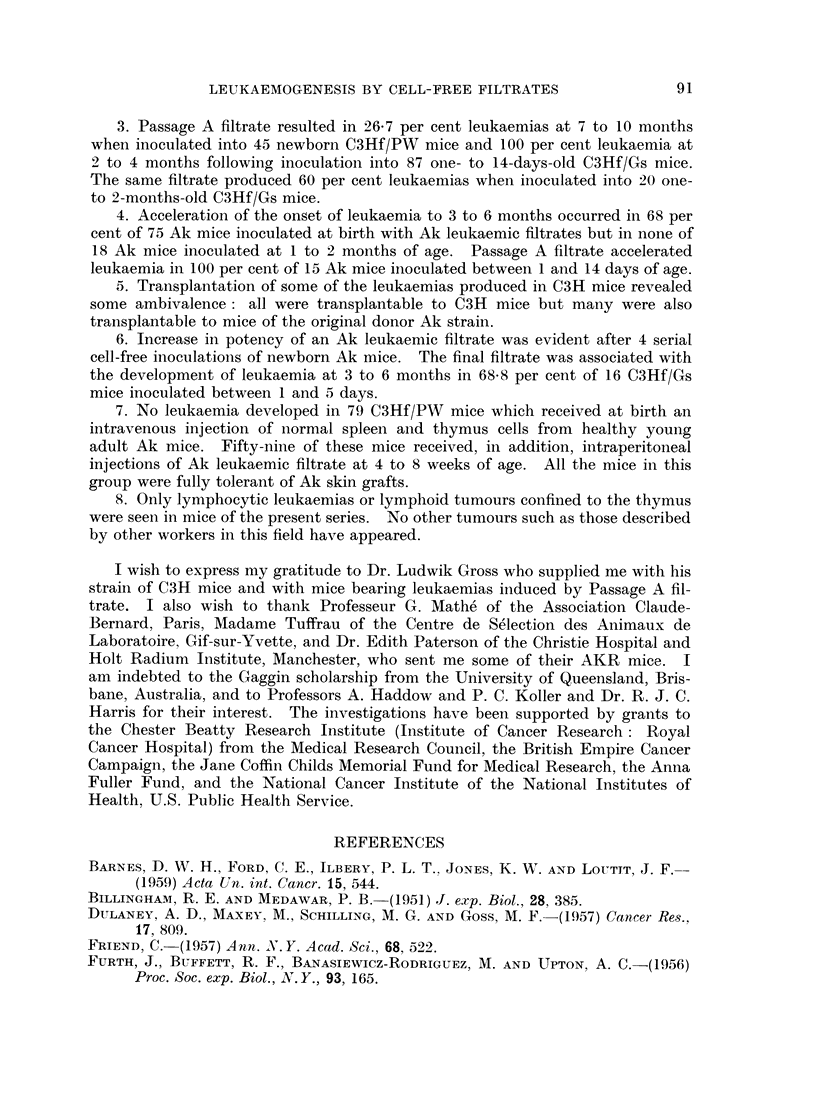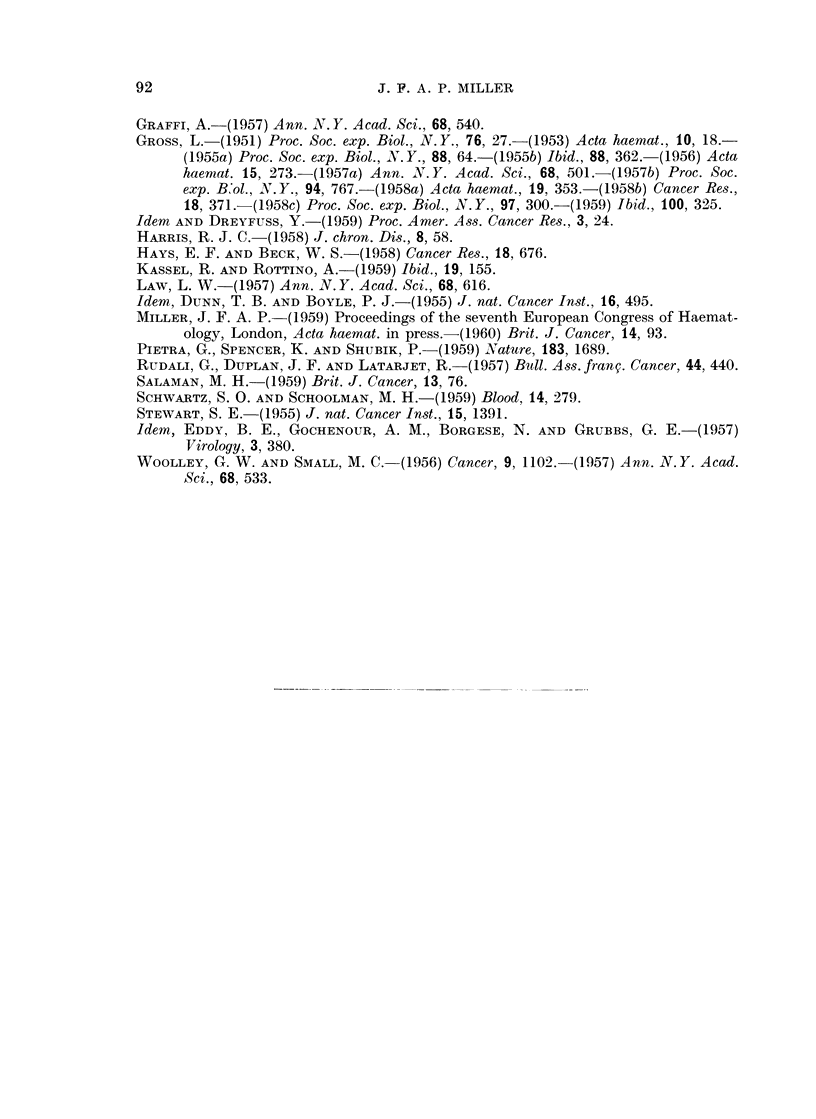# Studies on Mouse Leukaemia. Leukaemogenesis by Cell-free Filtrates Inoculated in Newborn and Adult Mice

**DOI:** 10.1038/bjc.1960.10

**Published:** 1960-03

**Authors:** J. F. A. P. Miller


### STUDIES ON MOUSE LEUKAEMIA. LEUKAEMOGENESIS BY CELL-FREE FILTRATES INOCULATED IN NEWBORN AND ADULT MICE

J. F. A. P. MILLER

*From the Chester Beatty Research Institute, Institute of Cancer Research : Royal Cancer Hospital, Fulham Road, London, S.W.3*

Received for publication December 11, 1959

FILTERED extracts, usually from neoplastic tissues, can induce leukaemias in newborn or adult mice of certain strains (Gross, 1957*a* ; Graffi, 1957 ; Friend, 1957 ; Schwartz and Schoolman, 1959). Gross has shown that such extracts from tissues of high-leukaemic strain mice with spontaneous lymphocytic leukaemias, when injected at birth into mice of low-leukaemic strains, will ultimately produce leukaemia in about a third of the recipient mice (Gross, 1958*b*). The resulting leukaemias were transplantable by cell graft in most cases to adult mice of the recipient line but only rarely to the donor strain in which the original leukaemia occurred.

Only newborn mice less than 16 hours after birth were found initially to be susceptible to the leukaemogenic activity of the extracts. When, however, the extracts were passed serially through several generations of susceptible mice, their potency was increased and young adult mice could be successfully inoculated (Gross, 1958*c*). Marked inter- and intra-strain sensitivity has been found in the response of the mice to inoculation of the extracts. In the C3H strain, for instance, the C3Hf/Gs subline is the most susceptible (Woolley and Small, 1957) while the C3Hf/An subline is hardly sensitive (Gross, 1955*a*). The necessity of using newborn mice and the marked differences in strain and substrain susceptibility have suggested the possibility that the disease only develops in mice that can acquire immune tolerance to the leukaemic agent (Harris, 1958 ; Barnes *et al.*, 1959).

A series of investigations has been commenced in this laboratory in an attempt to elucidate the mechanism of leukaemogenesis by cell-free extracts of leukaemic tissues. The present paper reports the results obtained in four different strains of mice inoculated as newborn or as adults with filtered extracts. A further paper is concerned with the role of the thymus in leukaemogenesis by cell-free filtrates of leukaemic tissues (Miller, 1960). A preliminary account of these studies has already been given (Miller, 1959).

### MATERIALS AND METHODS

*Mice.*—Experiments were carried out on mice of four different inbred strains (Table I). Inbreeding in mice of all four strains has been maintained by strict sib-matings in our laboratory at Pollards Wood since the time of their acquisition.

*Filtrates.*—Cell-free filtrates were prepared from freshly obtained tissues by a method closely following that of Gross (1958*b*). The tissues were homogenized in

TABLE I.—*Source and Date of Acquisition of Inbred Strains*

| Strain | Date acquired | Source |
|---|---|---|
| C3Hf/PW | 1938 | Bittner. |
| C3Hf/Gs | 1958 | Gross via Woolley. |
| CBA/H | 1949 | Harwell. |
| $Ak_i$ | 1945 | Furth via Engelbreth-Holm. |

ice-cold saline with a previously chilled mortar and pestle so as to make a 20 per cent homogenate. This was centrifuged in a Servall centrifuge at 1400 *g* for 15 minutes in the cold room. The supernatant fluid was then centrifuged at 7000 *g* for 10 minutes and the final supernatant fluid filtered through a chilled Selas 02 porosity filter candle under vacuum pressure. The extracts were either used immediately or sealed in 2 ml. glass ampoules and stored at −79° C.

The extracts were prepared from thymus, spleen and lymph nodes of mice with either spontaneous or induced leukaemias: the spontaneous leukaemias were obtained from our own colony of $Ak_i$ and from AKR mice received from other laboratories; the induced leukaemias were obtained from C3Hf/Gs mice inoculated by Dr. Gross with his potent Passage A filtrate (Gross, 1957*b*).

*Inoculation procedures.*—Filtrates were generally injected into newborn mice less than 16 hours after birth, each baby mouse receiving 0·1 ml. by the intraperitoneal route, the needle first traversing the thigh muscles to avoid leakage. When Passage A filtrate was used however, suckling mice up to 14 days of age were injected, the volume of the inoculum being varied according to age: at 1 and 2 days 0·1 ml. was given, at 3 and 4 days 0·2 ml. and at 5 to 14 days 0·3 ml. Adult mice inoculated with filtrates received 0·5 ml. intraperitoneally daily for 3 days. Control mice were usually littermates identified by tail-clipping; they received either saline of filtrates heated to 56° C. for 30 minutes.

*Transplantation of leukaemias.*—For transplantation of leukaemias, cell suspensions were prepared from fresh leukaemic spleen by teasing out with forceps in a dish of saline. The cells were washed in saline to the desired volume so that 0·5 ml. of the suspension contained 30 to 50 million cells. This amount was injected intraperitoneally into adult mice of the strains tested for transplantability. The cells of each case of leukaemia to be transplanted were injected into 3 to 5 mice of each strain.

*Induction of immune tolerance.*—For induction of specific immune tolerance to Ak tissues, cell suspensions from thymus or spleen of one-month-old healthy $Ak_i$ donors were prepared by teasing out in buffered Ringer phosphate solution, washing twice, and resuspending in the buffered solution so that 0·05 ml. of the suspension contained 5 to 7 million cells. This amount of the freshly prepared suspension was injected into the anterior facial vein of newborn C3Hf/PW mice less than 20 hours after birth.

*Skin grafts.*—Skin grafts were performed according to the method of Billingham and Medawar (1951) at 6 to 8 weeks of age. The grafted skin was from 2-months-old healthy $Ak_i$ female mice and served as the external indicator of the tolerant state. Only fully tolerant mice, carrying healthy skin grafts were used experimentally.

*Histology.*—Tissues for histological examination were fixed in Bouin's fluid and stained in haematoxylin and eosin.

### RESULTS

Detailed results are presented in Tables II to VIII. The salient features of these tables may be summarized as follows.

*Incidence of spontaneous leukaemia* (Table II)

Only the $Ak_i$ strain had a high incidence of spontaneous leukaemia during the fourteen month period of observation. The final figures for the full life span of the low-leukaemic strains may well be rather different.

TABLE II.—*Incidence of Spontaneous Lymphocytic Leukaemia in Untreated Mice of Four Different Strains*

| Strain of mice | Number of mice observed for 14 months | Mice with lymphocytic leukaemia: Number | Age in months | Per cent |
|---|---|---|---|---|
| C3Hf/PW | 227 | 2 | (12, 14) | Less than 1 |
| C3Hf/Gs | 79 | 0 | — | 0 |
| CBA/H | 121 | 0 | — | 0 |
| $Ak_i$ | 193 | 171 | Average 8·9 | 88 |

*Incidence of lymphocytic leukaemia in mice inoculated at birth with cell-free extracts of leukaemic tissues* (Table III)

(a) *Controls*.—The saline and heat-inactivated filtrate controls in all cases did not have a significantly different incidence of leukaemia from the completely untreated mice.

TABLE III.—*Incidence of Lymphocytic Leukaemia in Mice Following Inoculation at Birth of Cell-Free Extracts*

| Strain | Filtrate given at birth | Number in group | Mice with lymphocytic leukaemia*: Number | Age in months | Per cent |
|---|---|---|---|---|---|
| C3Hf/PW | Ak leukaemic | 68 | 8 | 7–14 (average 11) | 11·8 |
| | Passage A | 45 | 12 | 7–10 (average 9) | 26·7 |
| | Saline | 39 | 0 | — | 0 |
| C3Hf/Gs | Passage A | 87† | 87 | 2–4 (average 3) | 100 |
| | Heated Passage A | 15 | 0 | — | 0 |
| CBA/H | Ak leukaemic | 71 | 5 | 6–15 (average 11) | 7 |
| | Saline | 40 | 0 | — | 0 |
| $Ak_i$ | Ak leukaemic | 75 | 51<br>8 | 3–6 (average 4·7)<br>7–12 | 78·7 |
| | Passage A | 15† | 15 | 3–6 (average 4·1) | 100 |
| | Saline | 34 | 31 | 7–11 (average 9·6) | 91 |

* Survivors are over 12 to 14 months of age.
† These mice were injected 1 to 14 days after birth.

(b) *Filtrates from Ak leukaemic tissues.*—Filtrates from leukaemic Ak mice had a marked effect only in $Ak_i$ mice where the average age of onset of the disease was reduced from about 9 months to about 5 months. This effect must be regarded as an acceleration of the leukaemogenic process. The total incidence of the disease in the inoculated group was slightly lower than in the completely untreated group as a result of a higher mortality from non-leukaemic causes in inoculated mice.

A slightly increased incidence of leukaemia was observed in C3Hf/PW and CBA/H mice following injection of Ak leukaemic filtrates at birth.

Thirty-one separate Ak filtrates were used in these experiments but only 8 were associated with leukaemogenic activity in C3Hf/PW and CBA/H mice (Table IV). Five filtrates each produced leukaemia in 2 mice and in 3 cases the 2 mice came from the same litter. Sixteen of the filtrates used were responsible for the development of early leukaemias in inoculated $Ak_i$ mice.

(c) *Passage A filtrate.*—Injection of Passage A filtrate at or soon after birth gave 100 per cent incidence of leukaemia in both C3Hf/Gs and $Ak_i$ mice as early as 2 to 4 months. This filtrate increased the incidence of leukaemia following injection into newborn C3Hf/PW mice to about 27 per cent the disease developing between 7 and 10 months of age.

TABLE IV.—*Activity of Filtrates from Tissues of Ak Mice with Spontaneous Leukaemia*

| | Recipients (inoculated at birth) | | | | | | | | | |
|---|---|---|---|---|---|---|---|---|---|---|
| | Low-leukaemic C3Hf/PW and CBA/H mice | | | | | High-leukaemic $Ak_i$ mice | | | | |
| | | | | Leukaemia incidence | | | | | Incidence of early (3-6 months) leukaemia | |
| Source of filtrates | Number of active filtrates | Number of mice inoculated | Number of susceptible litters | Number | Per cent | Number of active filtrates | Number of mice inoculated | Number of susceptible litters | Number | Per cent |
| $Ak_i$ (Pollards Wood) | 5/15 | 73 | 6/26 | 7 | 9·6 | 10/14 | 40 | 15/18 | 27 | 67·5 |
| AKR (Manchester) . | 1/8 | 32 | 2/10 | 2 | 6·3 | 3/5 | 17 | 3/5 | 11 | 64·7 |
| AKR (Gif-sur-Yvette) | 1/4 | 19 | 1/4 | 2 | 10·5 | 2/3 | 9 | 2/3 | 7 | 77·8 |
| AKR (Paris) . . | 1/4 | 15 | 1/4 | 2 | 13·3 | 1/2 | 9 | 2/3 | 6 | 66·7 |
| Total . . | 8/31 | 139 | 10/44 | 13 | 9·4 | 16/24 | 75 | 22/29 | 51 | 68·0 |

*Transplantability of the leukaemias*

Seven of the leukaemias produced in C3Hf/PW after injection of filtrates of Ak leukaemic tissues were tested for transplantability in $Ak_i$ and C3Hf/PW mice. All were transplantable to C3Hf/PW and 3 to Ak mice as well. Twenty-four Passage A induced leukaemias in C3Hf/Gs mice were transplanted. All were transplantable to C3Hf/Gs and 15 also to $Ak_i$. Three leukaemias arising in CBA/H mice were transplantable only to CBA/H mice.

*The development of a potent Ak filtrate* (Table V)

Filtrates from Ak leukaemic tissues was passed through serial cell-free inoculations into 4 successive generations of newborn Ak mice. In each passage, the first Ak mouse that developed leukaemia was used as donor for the preparation

TABLE V.—*The Incidence of Lymphocytic Leukaemia in C3Hf/Gs Mice Following Inoculation of Ak Leukaemic Filtrates Passaged Serially Through Ak Mice*

| Filtrate given at birth | Number of C3Hf/Gs mice inoculated | C3Hf/Gs mice with lymphocytic leukaemia: Number | Age in months | Per cent |
|---|---|---|---|---|
| Ak leukaemic (from spontaneous case) | 14 | 0 | — | 0 |
| Ak leukaemic (after 3 passages in Ak mice) | 11 | 5 | 4–10 (average 7·4) | 45·5 |
| Ak leukaemic (after 4 passages in Ak mice) | 16* | 11 | 3–6 (average 5·1) | 68·8 |

* These mice were inoculated between 1 and 5 days after birth.

of the next passage-extract. The final filtrate considerably increased the total incidence of the disease and accelerated the average age of onset when inoculated into C3Hf/Gs mice at or soon after birth.

TABLE VI.—*Incidence of Lymphocytic Leukaemia in Mice Following Inoculation of Cell-Free Extracts at 4 to 8 Weeks of Age*

| Strain | Filtrate given at 4 to 8 weeks | Number in group | Mice with lymphocytic leukaemia*: Number | Age in months | Per cent |
|---|---|---|---|---|---|
| C3Hf/PW | Ak leukaemic | 34 | 0 | — | 0 |
| C3Hf/Gs | Ak leukaemic | 18 | 0 | — | 0 |
| | Passage A | 20 | 12 | 4–8 (average 6·8) | 60 |
| $Ak_i$ | Ak leukaemic | 18 | 15 | 8–11 (average 8·9) | 83·3 |

* Survivors are over 12 to 14 months of age.

*The incidence of lymphocytic leukaemias in mice inoculated as adults with cell-free extracts of leukaemic tissues* (Table VI)

Cell-free filtrates of leukaemic tissues of Ak mice were not capable of inducing leukaemias in adult C3Hf/Gs or C3Hf/PW mice. Though a high number of $Ak_i$ mice developed leukaemia following injection at 4 to 8 weeks of age with filtrates of Ak leukaemic tissues, the total incidence and average age of onset were almost the same as in untreated $Ak_i$ mice. It must, therefore, be assumed that the filtrates had no noticeable effect when injected into adult mice.

Passage A filtrate given to C3Hf/Gs at 4 to 8 weeks of age did produce some leukaemias but not as efficiently as the same filtrate given at or soon after birth.

*The incidence of lymphocytic leukaemia in C3Hf/PW mice tolerant to $Ak_i$ and inoculated as adults with cell-free extracts of Ak leukaemic tissues* (Table VIII)

An intravenous injection of healthy $Ak_i$ spleen or thymus cells into newborn C3Hf/PW mice induced long-lasting tolerance to $Ak_i$ skin grafts in up to 90 per cent of the mice (Table VII). Transplants of Ak leukaemic cells grew progressively in all cases in tolerant C3Hf/PW mice but never in non-tolerant mice. On further transplantation to groups of non-tolerant C3H and of $Ak_i$ mice the trans-

TABLE VII.—*Tolerance to Ak Skin Graft in C3Hf/PW Mice Inoculated at Birth with Ak Spleen or Thymus Cells*

| Ak cells given at birth | | Number of mice in group | Number of mice fully tolerant |
|---|---|---|---|
| Number | Type | | |
| 5–7 million | Spleen | 143 | 128 (89%)* |
| 5–7 million | Thymus | 161 | 137 (85%)* |
| 0 | — | 57 | 0 (0%)† |

* Ak skin graft intact to date (over 12 months).
† Ak skin graft rejected in 11 $\pm$ 1 days.

plants were successfully established only in Ak mice thus showing that the genetic constitution of the transplanted leukaemia was still Ak.

TABLE VIII.—*Incidence of Lymphocytic Leukaemia in C3Hf/PW Mice Tolerant to Ak Following Inoculation of Ak Leukaemic Extracts at 4 to 6 Weeks of Age*

| Intravenous injection at birth | Filtrate given at 4 to 6 weeks | Number of mice in group | Mice with leukaemia | | |
|---|---|---|---|---|---|
| | | | Number | Age | Per cent |
| Ak spleen cells | Ak leukaemic | 31 | 0 | — | 0 |
| Ak thymus cells | ,, ,, | 28 | 0 | — | 0 |
| Ak spleen or thymus cells | None | 20 | 0 | — | 0 |

No leukaemia occurred in C3Hf/PW mice made tolerant to Ak and injected 1 month after birth with cell-free filtrates from Ak leukaemic tissues (Table VIII). Sixteen of the mice in this group received at least one of the 8 filtrates that was associated with leukaemogenic activity following inoculation into newborn C3Hf/PW or CBA/H mice.

### DISCUSSION

The work described here confirms the results obtained by Gross (1958*b*) and others (Woolley and Small, 1956 ; Furth *et al.*, 1956 ; Dulaney *et al.*, 1957 ; Hays and Beck, 1958 ; Kassel and Rottino, 1959).

Cell-free extracts of tissues of Ak mice with spontaneous leukaemia are clearly capable of leukaemogenic activity. In the high-leukaemia Ak strain and in the (AKRXC3Hf)$F_1$ hybrids, there is little or no change in leukaemia incidence but an acceleration of the onset of the disease following inoculation of extracts at birth (Rudali, Duplan and Latarjet, 1957 ; Law, Dunn and Boyle, 1955). The leukaemic agent has been shown to be present in young healthy Ak mice (Gross, 1951, 1953), so that inoculation of leukaemic extracts into these mice must be assumed to increase the quantity of agent already present in the host. Acceleration of leukaemia may thus be explained on a purely quantitative basis. Extracts from normal tissues of low-leukaemic C3H mice failed to be associated with leukaemogenic activity following inoculation into newborn C3H mice (Gross, 1955*b*). These mice may, therefore, not carry the leukaemic agent, and leukaemia occurring as a result of inoculation of Ak leukaemic extracts must presumably have been induced by the agent. It is true, however, that the disease occurs spontaneously in low-leukaemic strain mice (Law, 1957) and two cases have been diagnosed in our colony of C3Hf/PW kept under observation for only 14 months.

These two cases still have to be explained. It is possible that some of the C3H mice carry the agent, but that our methods are not sensitive enough to demonstrate its presence ? If this is so, does the inoculation of leukaemic filtrate at birth simply accelerate the onset of the disease in mice that would develop it if they lived long enough ? Some agent must be present in C3H mice since Gross claims to have activated it by X-irradiation (Gross, 1958*a*). The point at issue is whether this agent is or is not identical to that present in high-leukaemia strains.

Filtrates from Ak leukaemic tissues have been shown to vary considerably in their activity. Many of them were associated with the development of early leukaemia in Ak mice, but only few produced leukaemia in C3Hf/PW mice (Table IV). Gross developed his potent Passage A filtrate (Gross, 1957*b*) by selecting an active Ak leukaemic extract and passing it through serial cell-free inoculation of newborn C3Hf/Gs mice. In the work reported here, a potent extract was obtained in a similar way but in a relatively shorter period of time, the extracts being passed through successive generations of newborn Ak mice instead of C3Hf/Gs mice (Table V). No doubt this procedure must select and concentrate active agent, since dilution of the final potent extract reduces the total incidence and delays the average age of onset of leukaemia in inoculated mice (Gross and Dreyfuss, 1959).

Subline differences exist in respect to susceptibility to leukaemogenesis by any given extract as was pointed out by Gross (1955*a*) and Woolley and Small (1957). In this study, a valid comparison can be made of the response of 2 sublines of C3H mice, both originally obtained from Bittner, since both were injected simultaneously with the same filtrate, Passage A. It is obvious from Table III that the C3Hf/Gs subline is by far the more sensitive.

Only newborn mice were susceptible to the leukaemogenic activity of filtrates prepared from tissues of Ak mice with spontaneous leukaemia. This may be due to :

(1) A state of immunological unresponsiveness ;

(2) a concentration effect ;

(3) factors, other than immunological, inherent to the particular stage of development of the animals at birth.

(1) It has been suggested that immunological tolerance is the reason why only newborn mice are susceptible to an agent recently isolated from a foreign strain (Harris, 1958 ; Barnes *et al.*, 1959). In this study, a state of acquired tolerance in C3H mice to transplantation antigens of Ak mice did not appear to give tolerance to the Ak leukaemic agent (Table VIII). The mice were fully susceptible to grafts of Ak leukaemic cells but not to the leukaemogenic activity of the Ak agent. For the induction of tolerance, normal Ak cells, which according to Gross (1953, 1959) contain the agent, were injected intravenously at birth, and yet no leukaemia developed. Perhaps the quantity of agent in 5 million normal Ak cells is not sufficient for leukaemogenesis, or perhaps the agent is intracellular, antigenically distinct and must be introduced in the free state in newborn animals.

(2) Simple quantitative factors may serve to explain the age susceptibility to the leukaemogenic effect of cell-free filtrates. For any active filtrate, the concentration of agent present in the newborn after inoculation must be relatively far greater than that which can be attained in an adult mouse. Augmentation of " infectivity " presumably occurs as a result of selection of the most active extract after serial passage through successive generations of mice of any one strain. A far greater concentration of active agent can then be achieved following inoculation into suckling or young adult mice. This may explain why acceleration of leukaemia in Ak mice by Ak filtrate can only occur when the filtrate is given at birth, and why, on the other hand, Passage A can accelerate Ak leukaemia when given to older Ak mice or can produce leukaemia after inoculation into young adult C3Hf/Gs mice.

(3) Shubik has pointed out that the response of newborn mice to filtrates should also be viewed as a response of a sensitive biological system to carcinogenesis (Pietra, Spencer and Shubik, 1959). He reports a high incidence of early malignant lymphomas in a relatively insusceptible strain of mice following the inoculation at birth of a single low dose of chemical carcinogen.

All the leukaemias developing in our inoculated mice were lymphoid with thymus involvement resembling typical spontaneous lymphomas in Ak mice. They were all transplantable to mice of the recipient line, and many were transplantable to both donor and recipient strains. This confirms similar observations made by Furth *et al.* (1956) and Gross (1958*c*). No other tumours, nor tumours characteristic of polyoma virus infection (Stewart *et al.*, 1957) were seen in any of the mice of the present series in contrast to the results of other workers in this field (Stewart, 1955 ; Law *et al.*, 1955 ; Salaman, 1959). Salaman, for instance, recorded the occurrence of 40 tumours (but no leukaemia) among 15 out of 23 C3Hf/Bi mice inoculated at birth with leukaemic filtrate. However, the filtrates were prepared from leukaemias that had already been transplanted three times by cell graft through AKR mice. Gross has stressed that as far as leukaemogenesis by cell-free extracts is concerned, the source of material should either be a spontaneous leukaemia or one induced with leukaemic extracts rather than a transplanted leukaemia (Gross, 1956, 1958*b*). Some supporting evidence for this claim was obtained by Kassel and Rottino (1959) who showed that extracts prepared from transplanted AKR leukaemic tissues resulted in the development of parotid tumours, adrenal tumours, osteosarcomas and fibromyxomas but no leukaemia.

It would be pure speculation to attempt, on the basis of the present results, to identify the agent in leukaemic filtrates as a virus or as a genic complex derived from either neoplastic or potentially neoplastic cells. An increase in leukaemia incidence, *per se*, does not establish the existence of a virus-mediated mechanism. Final identification of the agent must await further investigations of its biological and physicochemical properties.

### SUMMARY

1. Newborn or adult mice of the low-leukaemic strains C3Hf/PW, C3Hf/Gs, CBA/H, and of the high-leukaemic strain $Ak_i$ were inoculated intraperitoneally with cell-free extracts prepared from tissues of Ak mice with spontaneous leukaemia, and of C3Hf/Gs mice with leukaemias induced by leukaemic filtrate, Passage A.

2. Ak leukaemic filtrates inoculated into newborn low-leukaemic strain mice resulted in leukaemia in 11·8 per cent of 68 C3Hf/PW mice at 7 to 14 months and 7 per cent of 71 CBA/H mice at 6 to 15 months. No leukaemia occurred when these filtrates were inoculated into 34 adult C3Hf/PW mice or 18 adult C3Hf/Gs mice.

3. Passage A filtrate resulted in 26·7 per cent leukaemias at 7 to 10 months when inoculated into 45 newborn C3Hf/PW mice and 100 per cent leukaemia at 2 to 4 months following inoculation into 87 one- to 14-days-old C3Hf/Gs mice. The same filtrate produced 60 per cent leukaemias when inoculated into 20 one- to 2-months-old C3Hf/Gs mice.

4. Acceleration of the onset of leukaemia to 3 to 6 months occurred in 68 per cent of 75 Ak mice inoculated at birth with Ak leukaemic filtrates but in none of 18 Ak mice inoculated at 1 to 2 months of age. Passage A filtrate accelerated leukaemia in 100 per cent of 15 Ak mice inoculated between 1 and 14 days of age.

5. Transplantation of some of the leukaemias produced in C3H mice revealed some ambivalence : all were transplantable to C3H mice but many were also transplantable to mice of the original donor Ak strain.

6. Increase in potency of an Ak leukaemic filtrate was evident after 4 serial cell-free inoculations of newborn Ak mice. The final filtrate was associated with the development of leukaemia at 3 to 6 months in 68·8 per cent of 16 C3Hf/Gs mice inoculated between 1 and 5 days.

7. No leukaemia developed in 79 C3Hf/PW mice which received at birth an intravenous injection of normal spleen and thymus cells from healthy young adult Ak mice. Fifty-nine of these mice received, in addition, intraperitoneal injections of Ak leukaemic filtrate at 4 to 8 weeks of age. All the mice in this group were fully tolerant of Ak skin grafts.

8. Only lymphocytic leukaemias or lymphoid tumours confined to the thymus were seen in mice of the present series. No other tumours such as those described by other workers in this field have appeared.

I wish to express my gratitude to Dr. Ludwik Gross who supplied me with his strain of C3H mice and with mice bearing leukaemias induced by Passage A filtrate. I also wish to thank Professeur G. Mathé of the Association Claude-Bernard, Paris, Madame Tuffrau of the Centre de Sélection des Animaux de Laboratoire, Gif-sur-Yvette, and Dr. Edith Paterson of the Christie Hospital and Holt Radium Institute, Manchester, who sent me some of their AKR mice. I am indebted to the Gaggin scholarship from the University of Queensland, Brisbane, Australia, and to Professors A. Haddow and P. C. Koller and Dr. R. J. C. Harris for their interest. The investigations have been supported by grants to the Chester Beatty Research Institute (Institute of Cancer Research : Royal Cancer Hospital) from the Medical Research Council, the British Empire Cancer Campaign, the Jane Coffin Childs Memorial Fund for Medical Research, the Anna Fuller Fund, and the National Cancer Institute of the National Institutes of Health, U.S. Public Health Service.

### REFERENCES

Barnes, D. W. H., Ford, C. E., Ilbery, P. L. T., Jones, K. W. and Loutit, J. F.—(1959) *Acta Un. int. Cancr.* **15**, 544.

Billingham, R. E. and Medawar, P. B.—(1951) *J. exp. Biol.*, **28**, 385.

Dulaney, A. D., Maxey, M., Schilling, M. G. and Goss, M. F.—(1957) *Cancer Res.*, **17**, 809.

Friend, C.—(1957) *Ann. N.Y. Acad. Sci.*, **68**, 522.

Furth, J., Buffett, R. F., Banasiewicz-Rodriguez, M. and Upton, A. C.—(1956) *Proc. Soc. exp. Biol., N.Y.*, **93**, 165.

Graffi, A.—(1957) *Ann. N.Y. Acad. Sci.*, **68**, 540.
Gross, L.—(1951) *Proc. Soc. exp. Biol., N.Y.*, **76**, 27.—(1953) *Acta haemat.*, **10**, 18.—(1955*a*) *Proc. Soc. exp. Biol., N.Y.*, **88**, 64.—(1955*b*) *Ibid.*, **88**, 362.—(1956) *Acta haemat.* **15**, 273.—(1957*a*) *Ann. N.Y. Acad. Sci.*, **68**, 501.—(1957*b*) *Proc. Soc. exp. Biol., N.Y.*, **94**, 767.—(1958*a*) *Acta haemat.*, **19**, 353.—(1958*b*) *Cancer Res.*, **18**, 371.—(1958*c*) *Proc. Soc. exp. Biol., N.Y.*, **97**, 300.—(1959) *Ibid.*, **100**, 325.
*Idem* and Dreyfuss, Y.—(1959) *Proc. Amer. Ass. Cancer Res.*, **3**, 24.
Harris, R. J. C.—(1958) *J. chron. Dis.*, **8**, 58.
Hays, E. F. and Beck, W. S.—(1958) *Cancer Res.*, **18**, 676.
Kassel, R. and Rottino, A.—(1959) *Ibid.*, **19**, 155.
Law, L. W.—(1957) *Ann. N.Y. Acad. Sci.*, **68**, 616.
*Idem*, Dunn, T. B. and Boyle, P. J.—(1955) *J. nat. Cancer Inst.*, **16**, 495.
Miller, J. F. A. P.—(1959) Proceedings of the seventh European Congress of Haematology, London, *Acta haemat.* in press.—(1960) *Brit. J. Cancer*, **14**, 93.
Pietra, G., Spencer, K. and Shubik, P.—(1959) *Nature*, **183**, 1689.
Rudali, G., Duplan, J. F. and Latarjet, R.—(1957) *Bull. Ass. franç. Cancer*, **44**, 440.
Salaman, M. H.—(1959) *Brit. J. Cancer*, **13**, 76.
Schwartz, S. O. and Schoolman, M. H.—(1959) *Blood*, **14**, 279.
Stewart, S. E.—(1955) *J. nat. Cancer Inst.*, **15**, 1391.
*Idem*, Eddy, B. E., Gochenour, A. M., Borgese, N. and Grubbs, G. E.—(1957) *Virology*, **3**, 380.
Woolley, G. W. and Small, M. C.—(1956) *Cancer*, **9**, 1102.—(1957) *Ann. N.Y. Acad. Sci.*, **68**, 533.